# Changes in caseload and the potential impact on surgical training: a retrospective review of one hospital's experience

**DOI:** 10.1186/1472-6920-6-6

**Published:** 2006-01-18

**Authors:** Iain Varley, James Keir, Phillip Fagg

**Affiliations:** 1 Doncaster & Bassetlaw Foundation Hospitals NHS Trust, Doncaster Royal Infirmary, Armthorpe Road, Doncaster, DN2 5LT

## Abstract

**Background:**

Recent reforms to the training grades have provoked debate about both quality and quantity of training. The bulk of previous research into this area has been qualitative, and little is known about the quantity of training opportunities. This study aimed to determine if the number of elective operations available to trainees was stable.

**Methods:**

The number of elective procedures carried out in each surgical specialty (General & Vascular Surgery, Urology, Orthopaedics, ENT) in a large district general hospital was analysed in 6 month periods and adjusted for the number of basic surgical trainees in each specialty. In order to allow comparison between specialties, results for each 6 month period were calculated as a percentage of those for the first period.

**Results:**

The number of elective operations available per trainee fell in 3 of the 4 specialties, with a rise in Orthopaedics. Overall, the number of operations available to each trainee was 56% of that less than a decade ago.

**Conclusion:**

The number of operations available in a conventional hospital setting is decreasing. Introduction of the Modernising Medical Careers reforms must take account of this if they are to succeed in improving the quality of surgical training.

## Background

Surgical training in the UK has undergone numerous reforms, from the Calman report to the implementation of the European Working Time Directive, which have combined to reduce length of training, normal working hours and exposure to out-of-hours emergency procedures [1,2] placing "serious challenges" on gaining sufficient experience[3].

The current Modernising Medical Careers[4] reforms seek to address this situation with the implementation of seamless training. The plans call for an initial broad base to training with a two-year foundation scheme, followed by specialist training of between 5 and 10 years depending on the specialty and concluding with the award of a Certificate of Completion of Training[5].

Previous studies have shown that trainees have less experience than their forebears, in both emergency and elective work [6,7] and that the impact of previous reforms has resulted in the majority (86.5%) of learning opportunities coming from surgical activity during normal working hours[8]. If training reforms are to be implemented appropriately, it is important to understand the nature of available resources in order to allow adequate planning of training.

In essence, are there enough operations available to trainees for the current reforms to succeed?

In an effort to answer this question, theatre records have been used to establish if the number of elective procedures in a large district general hospital has changed, and if this equates to a change in the number of training opportunities available to individual trainees.

## Methods

Numbers of basic surgical trainees in each specialty by sixth month periods were obtained from medical staffing records.

Theatre systems allowed identification of all elective procedures carried out during normal working hours between August 1996 and February 2004. These were allocated a specialty according to the named consultant, and totalled in six-month periods. Data for Orthopaedic lists was incomplete until February 1997. The number of procedures performed was then adjusted for the number of trainees in the relevant specialty at that time (number of operations in each specialty divided by number of trainees) to give a measure of the number of cases a trainee had the potential to attend. In order to enable comparison between specialties, the number of procedures available per trainee in each sixth month period was calculated as a percentage of the figure for the first sixth month period in that specialty.

## Results

The number of trainees in all specialties changed little over the study period (table 1). A total of 61329 operations were performed during the study period, with a fall from 1062 in the 6 months to February 1997 to 917 in the 6 months to August 2005 in general and vascular surgery, 499 to 340 in urology, 1605 to 1008 in ENT and a rise from 481 in the six months to August 1998 to 756 in the 6 months to August 2005 in orthopaedics.

**Figure 1:**
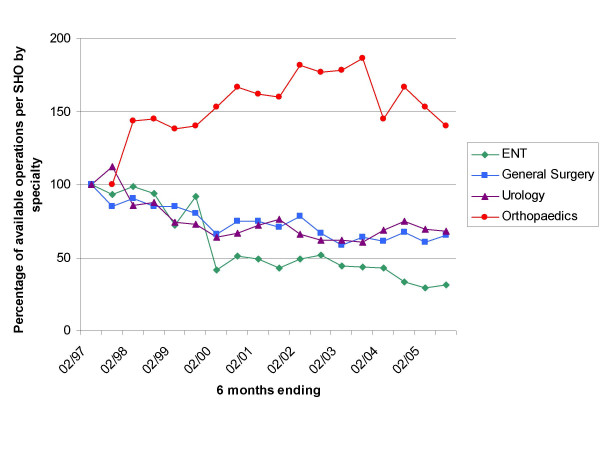
Changes in operations available per trainee by specialty.

**Figure 2:**
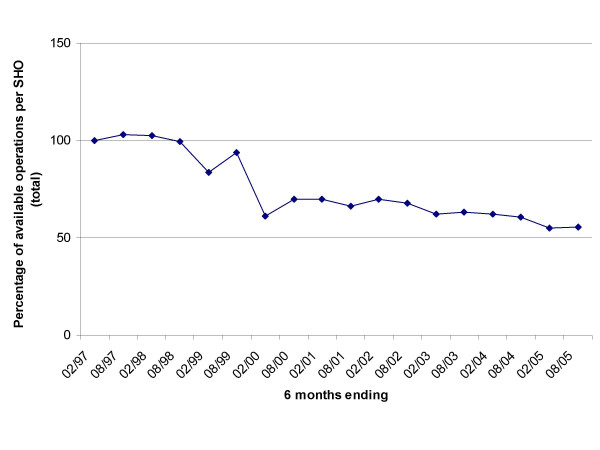
Changes in operations available per trainee across all specialties.

The number of elective operations available per trainee fell in 3 of the 4 specialties, to 31% in ENT, 65% in General and Vascular surgery, 68% in Urology, with a rise in Orthopaedics to 140% of those available in the first 6 month period (figure 1). Overall, the number of operations available to each trainee was 56% of that in 1996 (figure 2).

## Discussion

Training to be a surgeon is more than about learning to operate, with the need to attain adequate judgement, skills, technique and professionalism. However, a surgeon with excellent judgement who cannot operate is not a surgeon at all, and so basic technical skills remain a fundamental part of surgical training, and are core to the implementation of Modernising Medical Careers.

Previous research of the perceived importance of surgical skills has shown that generic tissue and instrument handling skills are the most valued technical aspects of a surgeon's abilities, both by consultants and those they train[9,10]. This supports the use of a broad based entry into training, particularly when it is considered that some specialties provide exceptional training in these basic skills, which are then transferable[11].

One potential area of concern with the introduction of seamless training is that if progress from the broad-based training to specialty training is not adequately assessed, it may result in passage to specialty training without acquisition of the basic skills necessary. The Modernising Medical Careers reforms attend to this by advocating a competence-based approach to assessment, and much previous research has focused on this aspect of surgical training, with several methods of assessing competency available[12,13].

In training, of course, there needs to be consideration of both quantity and quality, with initial acquisition of surgical skills which are then honed with experience. The bulk of the debate in terms of quantity of training has focussed on the reduction in hours of training following the widely-opposed introduction of the European Working Time Directive [14,15], and more recently the length of training[2]. Of particular concern has been the introduction of full-shift rotas, as these remove juniors from a proportion of normal working hours and threaten experience[15,16], the majority of which comes as programmed activity during the normal working day[8].

In theory, competency-based assessment should ensure trainees acquire the necessary skills prior to progressing but it can only succeed if sufficient experience is available to gain the basic skills in the first place. Those studies which have demonstrated a reduction in the number of procedures performed by trainees have not taken account of the number of operations available [6-8,17]. This study shows that the number available on a conventional training scheme is decreasing. As we are unable to say how many operations were either suitable for or used for training, this reduction would appear to be of little concern, as it would not necessarily impact upon the number of training cases available to juniors. However, as far back as 1997 there was already a paucity of training opportunities in comparison to the expectations of trainers[18] and in specialties where defined targets for experience exist, less than half of trainees are gaining the experience they require[17].

Any potential for further reduction to hands-on experience must therefore be closely guarded and compensated for.

The possible causes for the reduction in elective caseload seen are numerous, and in our unit include the introduction of a Day Case centre, and more recently Diagnosis and Treatment Centres and Independent Sector Treatment Centres, which will have resulted in loss of a proportion of elective work[19]. Other factors will impact upon a trainee's ability to make use of the opportunities, such as the proportion of time spent in non-clinical tasks[20,21]. Previous research has shown that workload has changed little despite the reduction in hours, resulting in a relatively higher workload for the hours worked[22], and that the majority of elective cases are currently performed by career-grade surgeons[23]. In addition, it can be argued that the increasing complexity of medicine has resulted in more non-clinical workload[24], particularly when day-case patients have been treated elsewhere, leaving patients with proportionally more co-morbidity for routine elective lists[19].

The operations may well be available, but the current arrangement of surgical training is providing diminishing access to them, and training reforms must take account of this if they are to succeed. Also, problems may be exacerbated as trainees with less experience become more senior, themselves then requiring training in procedures which would previously have been available to their juniors.

Such problems may be addressed primarily through better organisation and supervision[25]; through expanding use of day surgery & treatment centre facilities for training in minor and intermediate procedures [26,27]; through the introduction of dedicated training lists where possible [3] or by halting the implementation of full-shift rotas, possibly with the option of being "on-call for training"[1]. The use of, and level of experience that can be gained from, alternatives to "in theatre" training, such as the use of simulators, is controversial[28,29], but may provide an alternative to some current aspects of basic skills training[2].

Another potential solution is to reduce non-operative workload, thereby allowing trainees more time to attend theatre. The use of clinical assistants[30,31], and expanded roles for nurses[32] has the potential to considerably reduce the non-operative workload per trainee[31,33], although where nurses are being trained as surgical care practitioners the volume of procedures required for their training may impact heavily on the number remaining for surgical trainees[33].

Another solution is an increase in the number of trainees, and medical school places have been expanding to allow more doctors to be trained. However, any expansion in the number of trainees has the potential to decrease any individual's access to training opportunities, a point reinforced by the data from this study, where the largest reduction in available cases per trainee came in ENT (figure 1), a specialty which had a two-fold increase in the number of trainees during the study period (table 1).

## Conclusion

The majority of trainees' surgical experience comes from normal working hours, in particular elective work. The opportunity to gain experience from such work was below the levels acceptable to trainers in 1997, and may have diminished further.

This has ramifications both in terms of expectations of trainers and in total surgical experience.

If the Modernising Medical Careers reforms are to succeed in improving the quality of training, they must take account of the quantity of training opportunities available, and make maximum use of the remaining elective work.

## Abbreviations used

ENT – Ear, Nose, Throat and Head & Neck surgery

## Competing interests

The author(s) declare that they have no competing interests.

## Authors' contributions

IV conceived of the study, helped with data collection and analysis and drafted the manuscript. JK collected the bulk of the data and helped draft the manuscript

PF participated in the design and coordination of the study.

All authors read and approved the final manuscript.

## Pre-publication history

The pre-publication history for this paper can be accessed here:



## Figures and Tables

**Table 1:** The numbers of junior trainees and consultants at the beginning and end of the study by specialty

	6 months ending 02/97		6 months ending 08/05	
	SHOs	Consultants	SHOs	Consultants

General/Vascular surgery	6	7	7	11
Orthopaedics	8	5	9	8
Urology	2	2	2	4
ENT	3	4	6	9
